# Fast and accurate prediction of positive and negative urine cultures by flow cytometry

**DOI:** 10.1186/s12879-016-1557-4

**Published:** 2016-05-17

**Authors:** Bijan Moshaver, Foppie de Boer, Heidi van Egmond-Kreileman, Ellen Kramer, Coen Stegeman, Paul Groeneveld

**Affiliations:** Department of Clinical Chemistry, Isala Hospital, Zwolle, The Netherlands; Department of Internal Medicine/Infectious Diseases, Isala Hospital, Zwolle, The Netherlands; Department of Internal Medicine/Division of Nephrology, University Medical Center Groningen and University of Groningen, Groningen, The Netherlands

**Keywords:** Urinary tract infection, Microbiological diagnostics, Flow cytometry

## Abstract

**Background:**

Urinary tract infection (UTI) is a widespread infectious disease in humans. Urine culture, a huge workload in the microbiology laboratory, is still the standard diagnostic test for UTI, but most of the cultures are negative. A reliable screening method could reduce unnecessary cultures and quicken reporting of negative results.

**Methods:**

We evaluated the usefulness of a flow cytometry (FC) screening method in the prediction of positive urine culture to reduce the number of urine cultures. The urine specimens sent to the laboratory for culture were tested with the flow cytometer Accuri C6. FC bacterial counts were compared to standard urine culture results to assess the best cut-off values.

**Results:**

Two hundred nine urine samples were included, of which 79 (37.8 %) were culture positive. On comparing the culture and the FC data in the ROC curve, the FC bacterial counts of ≥10^6^ bacteria/mL provided a reliable screening for bacteriuria with a sensitivity and specificity of 99 and 58 %, respectively. All negative FC results (<10^6^ bacteria/mL) showed a negative predictive value of 99 % with a negative likelihood ratio of 0.02. The FC bacterial counts of ≥10^8^/mL showed a positive predictive value of 99 % with a positive likelihood ratio of 60.9.

**Conclusions:**

Counting bacteria in human urine samples by the FC is a fast, accurate and cost-effective screening method for bacteriuria. Our results showed that FC is able to rule out UTI, which can lead to a substantial reduction (36 %) of urine cultures. It also demonstrated that this method predicts positive cultures accurately.

## Background

Urinary tract infection (UTI) is one of the most common infections in humans [1]. Urine culture is still the standard diagnostic test for UTI in a symptomatic patient, which additionally provides information about the pathogen and its antibiotic susceptibility [2]. However, it is a time-consuming and costly test in which a large number of cultures are negative.

Urinary dipstick testing for nitrite and leukocyte esterase is the most commonly used test to rule in UTI in the general practice. It is a fast and cheap method which can modestly improve diagnostic precision but poorly rule out UTI [3–5]. Therefore, clinicians have to take account of the poor negative predictive value (NPV) of this test and other strategies are needed. Other techniques such as microscopic examination of urine sediment and Gram-staining may have superior NPV, but are either subjective or labor-intensive and time-consuming [6–8].

Automated urinalysis by semi-flow cytometry (SFC) using Sysmex UF-1000i has been described as a fast and accurate technique to count bacteria in urine samples, which may result in a reduction of urine culture, labor and costs [9, 10]. The Sysmex UF-1000i is equipped with one excitation laser (633 nm) and uses forward scatter (FSC) and side scatter (SSC) for size and granularity determination plus two fluorescence parameters to detect bacteria. Flow cytometry (FC) that uses more than one excitation laser to detect multi fluorescence parameters, is rapidly becoming a routine methodology in microbial studies [11–13]. FC allows the examination of a large number of cells at a time, recording for each cell several different parameters that can later be linked to a wide variety of cellular characteristics [14].

As current tests are moderately reliable and fast, or more reliable but time-consuming, the urinalysis by FC could be a good alternative to rule out UTI in clinical decision making. Therefore, we aimed to assess the predictive value of FC urine bacterial count to predict positive and negative cultures. By using the flow cytometer Accuri C6, we developed a sensitive and rapid FC assay to quantify the total urine bacterial numbers. We hypothesized that bacteria counting with this technique could have a high value in the prediction of positive urine culture compared with regular urinalysis leading to a fast rule out of UTI. To validate this test we used the predictive value and compared the FC bacterial count to urine cultures at different cut-off values of colony-forming units per mL (CFU/mL). To our knowledge, this study is the first one to use FC to identify the independent predictive value of bacterial counts among patients with presumed UTI.

## Methods

This research was performed at the Department of Clinical Chemistry of the Isala Hospital in Zwolle, Netherlands. Urine samples of 209 patients with suspected UTI from general practitioners, outpatient and clinical departments were collected without any prior selection and sent to the Department of Medical Microbiology. Urine samples were then randomly selected and sent to the clinical chemistry laboratory. At arrival all consecutive samples were evenly divided over two aliquots. One aliquot was used for culture and the other one was kept at 4 °C and used within 24 h for FC bacterial counting in the clinical chemistry laboratory. The medical ethical committee of our hospital declared no objection and informed consent was not needed. The definition of positive urine cultures in patients with UTI is still a matter of debate [10], but most often ≥10^5^ CFU/mL is used to confirm urinary tract infection [4]. We used different cut-off values for positive urine cultures (≥10^3^, ≥10^4^ and ≥10^5^ CFU/mL) to evaluate the results of the FC analysis. Standard microbiological methods were used for semi-quantitative determination and identification of uropathogens [15].

### Flowcytometric urinalysis

All urine samples were analysed for bacterial count by the flow cytometer Accuri C6 (BD Biosciences, USA) in the clinical chemistry laboratory. The instrument was validated and maintained daily according to the user manual. The Accuri C6 is equipped with two excitation lasers: a blue solid state (488 nm) and a diode red (640 nm) providing up to 6 simultaneous detection parameters, including 4 fluorescent colors plus FSC and SSC. The accuracy of the bacterial counting by the Accuri C6 was first validated using Trucount-beads tubes (BD Biosciences, USA) and different dilutions (10×, 100× and 1000×) of a bacterial strain (Escherichia coli, ATCC 25922, BD Biosciences, France). The calculated bacterial counts with beads were compared with those without beads to evaluate the counting accuracy of the Accuri C6.

**Fig. 1:**
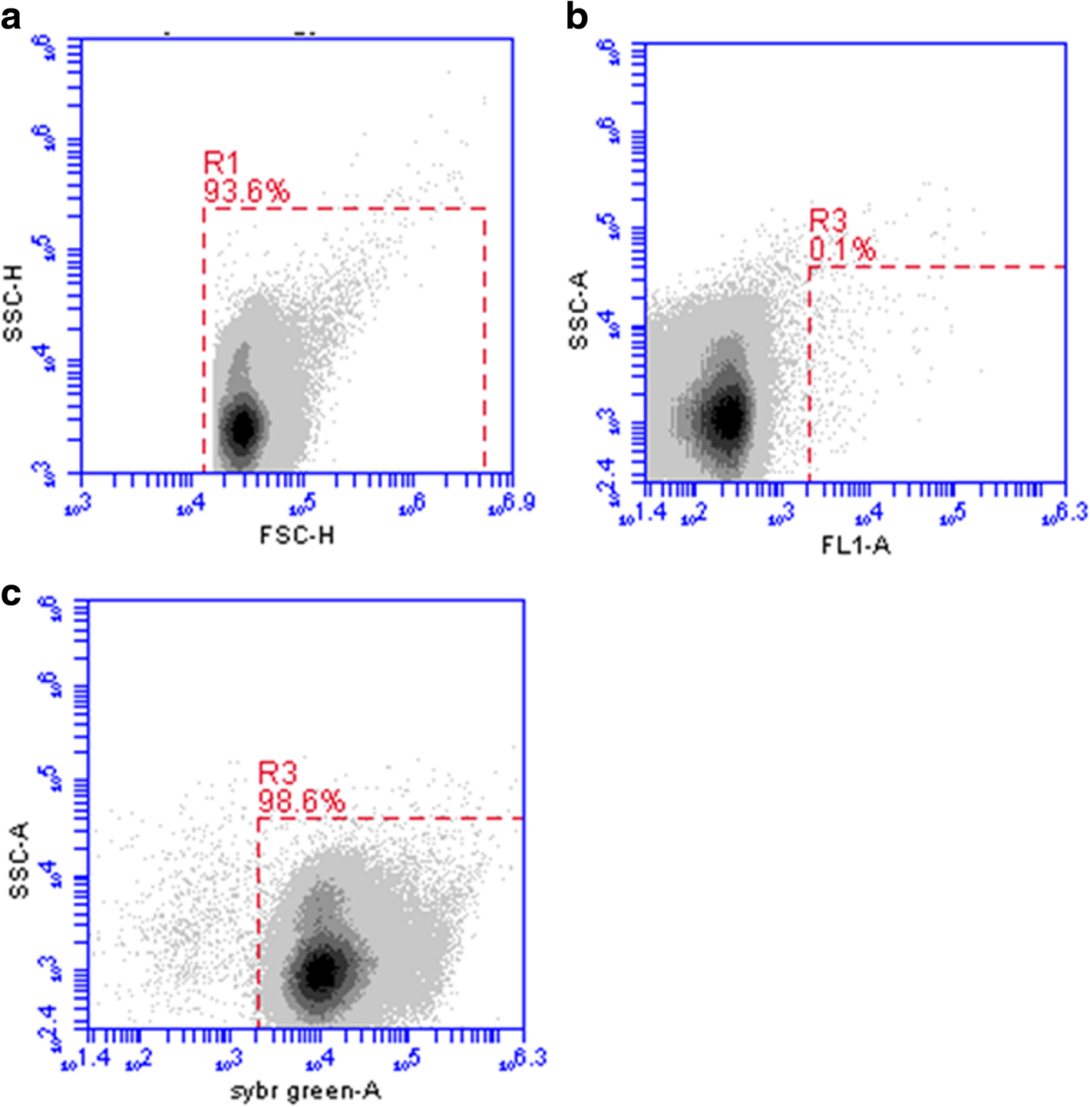
FC bacterial counting in urine samples. Bacteria were stained with SYBR Green and measured by the Accuri C6 as outlined in methods. **a**-**c** One representative example of urine bacterial counting. **a** FSC vs. SSC of bacteria detected in a urine sample (R1). Gate R1 is used in B and C. **b** FL-1 vs. SSC of an unstained urine sample as a negative control for SYBR Green (SG) staining. R3 gate shows the background staining (0.1 %). **c** SG vs. SSC of a stained urine sample. R3 gate shows the amount of SG-stained bacteria present in urine sample, which is used to calculate the absolute amount of bacteria per mL urine sample

The number of bacteria in human urine samples was assessed by FC using the following protocol: 5 mL of undiluted urine was washed once with staining buffer (phosphate-buffered saline/0.01 % Tween-20/1 mmol ethylene-diaminetetraacetic acid (EDTA), both from Sigma Life Science, USA) and centrifuged (5000 rpm, 5 min at room temperature) to reduce background noise. The pellet was resuspended in 5 ml staining buffer and then the washed urine was diluted 1 to 100 with staining buffer. Subsequently, 500 μl of diluted urine was added to 500 μl of “pre-diluted” (30000×) SYBR Green (Life Technologies, Invitrogen) and incubated for 10 min at 37 °C. SYBR Green (SG) has an excitation/emission maxima at 494 and 521 nm and binds to DNA of bacteria and can therefore be used to count bacteria. Next, a fixed volume (50 μl) of the urine SG-stained bacterial cells was analysed on the flow cytometer (Fig. 1) during 5 min using low sample rate and a selected threshold setting on FSC and SSC to discriminate bacterial cells from relatively large particles. The instrument threshold defines the minimum scatter needed to trigger an event that will be processed by the system software. It allows not only to reduce the electronic background noise but also to get rid of the unwanted non-target particles. Particles with low fluorescence and low SSC have a greater potential to interfere with the actual determination of the bacterial density, but these can easily be separated in the Green fluorescence plot as they do not stain with SG. Within a few minutes, the absolute amount of bacteria per mL could be calculated by multiplying the amount of counted SG-stained bacteria detected in the dot plot (gate R3 in Fig. 1) with the conversion factor (4000×); one representative example is shown in Fig. 1.

### Statistical analysis

Statistical analysis was performed using SPSS 22.0 software package. FC bacterial counts were compared to bacterial urine culture results with a bacteriuria at ≥10^5^ CFU/ml as most commonly used. In addition, we assessed the performance of FC counts at different cut-offs. At each cut-off point we determined the sensitivity, specificity, positive predictive value (PPV) and negative predictive value (NPV). Furthermore, positive and negative likelihood ratios (LR+ and LR-) were calculated. LR+ above 10 or LR- below <0.1 are considered to provide strong evidence to rule in or rule out diagnoses respectively [16]. We used a Receiver Operating Characteristic (ROC) curve to determine the best cut-off point with highest predictive rule out value defined by FC analysis. The area under the receiver operator curve (AUC) is the sum of the highest sensitivity and specificity and is expressed as mean plus 95 % confidence interval (Cl). Average values for the FC bacterial counts of the counting method were expressed as mean ± SEM.

## Results

The counting method of the Accuri C6 was validated using Trucount-beads. The results for counting methods (with and without beads) were similar: 67,483 ± 4291 (range 65,881–70,230) versus 70,394 ± 4396 (range 68,823–73,321) bacteria/mL with and without beads, respectively (*n* = 5). The Pearson correlation coefficient between the two methods was 0.986. So it could be concluded that FC bacterial counting by the Accuri C6 is an accurate and reliable method to quantify bacteria in urine samples.

We included different definitions of positive bacterial culture (≥10^3^, ≥10^4^ and ≥10^5^ CFU/mL) to show the influence on the sensitivity and specificity of the FFC bacterial counting method (Table 1). The diagnostic values of FC analysis at cut-off value of ≥10^5^ CFU/mL are summarized in Table 1A. Urine bacterial cultures at FC cut-off value of ≥10^5^ bacteria/mL were true-positive in 79 samples (38 %). We found 128 false-positive FC results, in which culture growth was less than 10^5^ CFU/ml and only 2 samples were true negative at this FC cut-off point.

**Table 1:** The value of FC urine bacterial count/mL in the prediction of positive urine culture at different cut-off points (*n* = 209)

Cut-off	TP	FP	TN	FN	Sensitivity	Specificity	PPV	NPV
A (≥10^5^ CFU/mL. *n* = 79)								
≥10^5^	79	128	2	0	100 %	2 %	38 %	100 %
≥10^6^	78	55	75	1	99 %	58 %	59 %	99 %
≥10^7^	65	9	121	14	82 %	93 %	88 %	90 %
≥10^8^	37	1	129	42	47 %	99 %	99 %	75 %
B (≥10^4^ CFU/mL. *n* = 100)								
≥10^5^	100	107	1	1	99 %	1 %	48 %	50 %
≥10^6^	91	42	66	10	90 %	61 %	68 %	87 %
≥10^7^	67	7	101	34	66 %	94 %	91 %	75 %
≥10^8^	37	1	107	64	37 %	99 %	97 %	63 %
C (≥10^3^ CFU/mL. *n* = 146)								
≥10^5^	146	61	0	2	99 %	0 %	71 %	0 %
≥10^6^	117	16	45	31	79 %	74 %	88 %	59 %
≥10^7^	73	1	60	75	49 %	98 %	99 %	44 %
≥10^8^	38	0	61	110	26 %	100 %	100 %	36 %

Diagnostic values when ≥10^5^ CFU/mL (A), ≥10^4^ CFU/mL (B), or ≥10^3^ CFU/mL (C) was chosen as cut-off value for positive urine cultures

*TP* true positive, *FP* false positive, *TN* true negative, *FN* false positive, *PPV* positive predictive value, *NPV* negative predictive value

At the cut-off point ≥10^6^ in the FC analysis, 78 samples are true-positive with 55 false-positive (FP) results while of 76 negative samples only one is false-negative (FN). Therefore, at this cut-off point sensitivity is 99 % and specificity is 58 % resulting in a strong NPV of 99 % and a LR- of 0.02, and this cut-off point essentially rules out a UTI, while the PPV is 59 %.

A FFC result of 10^7^ or higher showed 9 false-positives and 14 false-negatives. When using 10^8^ as cut-off, only one false-positive was found and the rest of the cultures were true positive, i.e., a PPV of 99 % and a high LR+ (60.9).

The FC results for other cut-off values for positive urine culture (≥10^4^ and ≥10^3^ CFU/mL) are shown in Table 1B and C, respectively. The FC analysis at both cut-off values for positive urine culture showed a lower sensitivity than that the FC analysis at higher numbers of CFU/mL (≥10^5^). The FC cut-off point ≥10^6^ showed a sensitivity of 90 and 79 % at 10^4^ CFU/mL (Table 1B) and 10^3^ CFU/mL (Table 1C), respectively. In contrast, the specificities were higher at this FC cut-off point compared with those at ≥10^5^ CFU/mL, i.e., 61 and 74 % at 10^4^ CFU/mL and 10^3^ CFU/mL, respectively.

Based on the FC results, UTI is very unlikely in patients with lower FC number of bacteria than 10^6^ and using this cut-off value a urine culture would only have been done in 133 of the 209 samples (64 %). Cultures are necessary in patients with a FC bacterial count of ≥10^7^ to confirm bacteriuria and to determine the resistance profile. Bacteriuria is very likely at FC bacterial count of higher than 10^8^, with a positive predictive value of 99 % and urine cultures are actually only necessary to determine the antibiotic resistance profile.

**Fig. 2:**
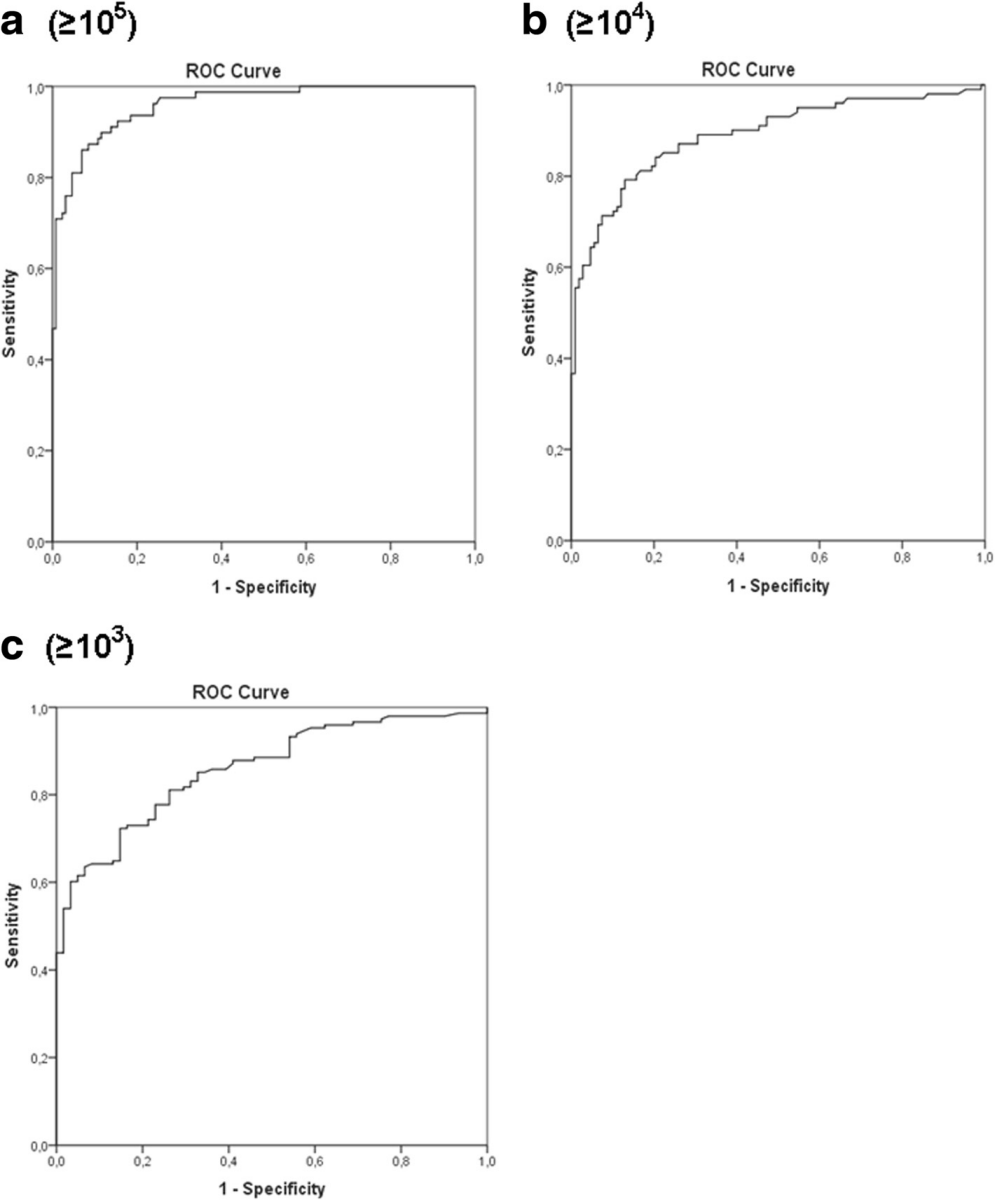
ROC curves for FC bacterial counting when ≥10^5^ CFU/mL (**a**), ≥10^4^ CFU/mL (**b**), or ≥10^3^ CFU/mL (**c**) was chosen as the definition for positive urine cultures

Moreover, we used a ROC-curve to calculate the exact cut-off point with highest sensitivity and specificity. The different ROC curves are shown in Fig. 2, showing the influence of the cut-off value of a positive urine culture on sensitivity and specificity of the FC bacterial counting method. Figure 2a shows that the AUC for bacterial detection by the FC method was 0.96 (95 % CI, 0.936–0.984) at ≥10^5^ CFU/mL. Using ≥10^5^ CFU/mL as cut-off for positive urine culture, we found a FC bacterial count of ≥10^6^ bacteria/mL as the best cut-off point for bacteriuria with a sensitivity and specificity of 99 % and 58 %. Using this cut-off value leads to 36 % reduction in urine cultures, missing only 1.2 % of positive cultures. All negative FC results (<10^6^ bacteria/mL) showed a negative predictive value of 99 % with a negative likelihood ratio of 0.02. The AUCs results for other cut-off values for positive urine culture (≥10^4^ and ≥10^3^ CFU/mL) are shown in Fig. 2b and c, respectively. Both cut-off values for positive urine culture showed a lower AUC value than the AUC at higher numbers of CFU/mL (≥10^5^). The AUCs for the FC method were 0.890 (95 % CI, 0,844–0,936) and 0.859 (95 % CI, 0.809–0.908) at ≥10^4^ and ≥10^3^ CFU/mL, respectively.

## Discussion

Urinalysis by SFC to detect bacteria has been described in the literature [9, 10]. However, little has been published on the application of FC in UTI diagnostics in clinical samples. Importantly, a highly sensitive test with a good NPV is needed in order to prevent unnecessary urine cultures. If urine cultures could be reduced by some 36 %, it means that a costs saving could possibly be achieved.

A survey in the literature about the application of the SFC Sysmex UF-1000i in bacteriuria screening showed a calculated sensitivity, specificity and AUC of 92 %, 60 % and 0.93, respectively [9, 10]. In our study high sensitivity is accompanied by high AUC (99 % and 0.96, respectively), compared to those found in other recent studies that evaluated the bacterial detection of the Sysmex UF-1000i. On the other hand, bacterial counting by this SFC instrument is automated which prevents mistakes, improve outcomes and simplify analysis [9, 10]. This is an advantage compared with the Accuri C6, which is not automated yet.

The definition of positive urine cultures in patients with UTI is still a matter of debate [10], but most often ≥10^5^ CFU/mL is used as the standard for diagnosis of UTI in patients with fever [4]. According to the Infectious Diseases Society of America (IDSA) guideline for uncomplicated UTI, ≥10^4^ CFU/mL can be considered as cut-off for positive culture [17]. Besides, ≥10^3^ CFU/mL has been be used as threshold for positive urine cultures in young women with uncomplicated UTI [18]. Furthermore, ≥10^3^ CFU/mL has been also marked as cut-off for positive bladder urine culture collected by suprapubic aspiration and urethral catheterization in patients with suspected UTI [19]. Although the diagnostic potential of the Accuri C6 is very high at cut-off value of ≥10^5^ CFU/mL, it is still quite good at level ≥10^4^ and ≥10^3^ CFU/mL showing a sensitivity of 90 and 79 %, respectively. In comparison, at ≥10^4^ and ≥10^3^ CFU/mL showed the Sysmex UF-1000i a sensitivity of 82 and 74 %, respectively [10]. Therefore, most of the clinically relevant infections can be diagnosed accurately using the Accuri C6. In our study the FC cut-off point ≥10^6^ essentially ruled out bacteriuria. As the FC urinalysis using SG-staining includes both viable and damaged bacteria, this could possibly explain the difference between cut-off values (≥10^5^ vs. ≥10^6^) found by culture and FC methods. Moreover, compared with the SFC automated method, the application of the FC method in UTI diagnostics may have additional advantages: The FC instruments contain more than one laser and they can provide at least 6 simultaneous detection parameters to measure accurately bacterial cell size, nucleic acid content and physiological state of each cell. Such characteristics allow a better resolution of different subpopulations within the mixed bacterial populations, and also an easy identification of particles that can interfere with the counts.

Classical microbiology techniques are relatively slow in comparison to other analytical techniques, as they require the isolation of the organism prior to identification and other possible testing. In most cases, culture results are available in 48 to 72 h. Life-threatening infections require prompt antimicrobial therapy and therefore need rapid and accurate diagnostic tests. FC allows bacteria detection in clinical samples in a rapid, flexible, and sensitive way. Bacteria can be identified on the basis of their peculiar cytometric parameters or by means of certain fluorochromes. Moreover, when properly applied, FC can be adjusted to use defined parameters that avoid subjectivity and aid the clinical microbiologist in the interpretation of specific results, particularly in the field of rapid diagnosis. Furthermore, FC devices have become user-friendly, the number of applications has been expanded, instrument software has been improved, and costs are gradually decreasing. However there are also some limitations to this technique in clinical microbiology laboratories: An important one is a lack of standardization in FC protocols. Standards are also lacking in how flow data are analyzed and reported, because of the massive amount of data generated. In addition, flow data analysis can become very complicated and relies almost exclusively on gating by a human expert. Besides, commercial kits to use this technique in the clinical microbiology laboratory are lacking. Finally, to optimize the cost-benefit ratio of FC in clinical microbiology laboratories, automation is necessary. This implies adapting flow cytometers to robotics for automatic sample analysis and developing software to automate analysis of the results.

Nevertheless, the use of FC in clinical microbiology is now more than a mere possibility. Today’s universal presence of flow cytometers should help clinical microbiologists to progressively incorporate FC into their standard protocols.

## Conclusion

To the best of the authors’ knowledge, this is the first study to demonstrate the predictive value of FC, which is much faster and cost-effective than the gold standard method, in the diagnostic procedure for UTI. Our result confirms the potential of FC urine bacterial counts to predict diagnosis: ‘Ruling out’ bacteriuria, leading to a substantial reduction of culture and ‘Ruling in’ bacteriuria, to confirm UTI and define the antibiotic resistance profile by culture.

### Ethics and consent to participate

The medical ethical committee of our hospital ruled that no formal ethics approval was required in this particular case. Informed consent was not needed, as we used the rest material and this was approved by the medical ethical committee of our hospital.

### Consent to publish

Not applicable.

### Availability of data and materials

They are archived in the digital database of the Department of Clinical Chemistry of the Isala Hospital.

## Abbreviations

UTI: urinary tract infection
FC: flow cytometry
SFC: semi-flow cytometry
FSC: forward scatter
SSC: side scatter
CFU: colony-forming units
EDTA: ethylene-diaminetetraacetic acid
SG: SYBR Green
TP: true positive
FP: false positive
TN: true negative
FN: false positive
NPV: negative predictive value
PPV: positive predictive value
LR+: positive and negative likelihood ratio
LR-: negative likelihood ratio
ROC: receiver operating characteristic
AUC: receiver operator curve
CI: confidence interval
IDSA: Infectious Diseases Society of America
